# Stochastic E2F Activation and Reconciliation of Phenomenological Cell-Cycle Models

**DOI:** 10.1371/journal.pbio.1000488

**Published:** 2010-09-21

**Authors:** Tae J. Lee, Guang Yao, Dorothy C. Bennett, Joseph R. Nevins, Lingchong You

**Affiliations:** 1 Department of Biomedical Engineering, Duke University, Durham, North Carolina, United States of America; 2 Institute for Genome Sciences and Policy, Duke University, Durham, North Carolina, United States of America; 3 Department of Molecular Genetics and Microbiology, Duke University Medical Center, Durham, North Carolina, United States of America; 4 Molecular and Metabolic Signalling Centre, St George's, University of London, London, United Kingdom; 5 Center for Systems Biology, Duke University, Durham, North Carolina, United States of America; Johns Hopkins University, United States of America

## Abstract

The transition of the mammalian cell from quiescence to proliferation is a highly variable process. Over the last four decades, two lines of apparently contradictory, phenomenological models have been proposed to account for such temporal variability. These include various forms of the transition probability (TP) model and the growth control (GC) model, which lack mechanistic details. The GC model was further proposed as an alternative explanation for the concept of the restriction point, which we recently demonstrated as being controlled by a bistable Rb-E2F switch. Here, through a combination of modeling and experiments, we show that these different lines of models in essence reflect different aspects of stochastic dynamics in cell cycle entry. In particular, we show that the variable activation of E2F can be described by stochastic activation of the bistable Rb-E2F switch, which in turn may account for the temporal variability in cell cycle entry. Moreover, we show that temporal dynamics of E2F activation can be recast into the frameworks of both the TP model and the GC model via parameter mapping. This mapping suggests that the two lines of phenomenological models can be reconciled through the stochastic dynamics of the Rb-E2F switch. It also suggests a potential utility of the TP or GC models in defining concise, quantitative phenotypes of cell physiology. This may have implications in classifying cell types or states.

## Abstract 2

A new, stochastic model of entry into the mammalian cell cycle provides a mechanistic understanding of the temporal variability observed across populations of cells and reconciles previously proposed phenomenological cell-cycle models.

## Author Summary

Mammalian cells enter the division cycle in response to appropriate growth signals. For each cell, the decision to do so is critically dependent on the interplay between environmental cues and the internal state of the cell and is influenced by random fluctuations in cellular processes. Indeed, experimental evidence indicates that cell cycle entry is highly variable from cell to cell, even within a clonal population. To account for such variability, a number of phenomenological models have been previously proposed. These models primarily fall into two types depending on their fundamental assumptions on the origin of the variability. “Transition probability” models presume that variability in cell cycle entry originates from the fact that entry in each individual cell is random but also governed by a fixed probability. In contrast, “growth-controlled” models assume that the growth rates across a population are variable and result in cells that are out of phase developmentally. While both kinds of models provide a good fit to experimental data, their lack of mechanistic details limits their predictive power and has led to unresolved debate between their practitioners. In this study, we developed a mechanistically based stochastic model of the temporal dynamics of activation of the E2F transcription factor, which is used here as a marker of the transition of cells from quiescence to active cell cycling. Using this model, we show that “transition probability” and “growth-controlled” models can be reconciled by incorporation of a small number of basic cellular parameters related to protein synthesis and turnover, protein modification, stochasticity, and the like. Essentially our work shows that each kind of phenomenological model holds true for describing a particular aspect of the cell cycle transition. We suggest that incorporation of basic cellular parameters in this manner into phenomenological models may constitute a broadly applicable approach to defining concise, quantitative phenotypes of cell physiology.

## Introduction

Cell-to-cell variability in the timing of cell-fate commitment is widely observed in biological settings [1]–[4]. In particular, the variable timing of transition from the quiescent to the proliferative state is a well-documented phenomenon [5]–[8]. In a population of proliferating cells, such variability is reflected in the partitioning of the population into subpopulations at various phases of the cell cycle. This phenomenon is observed even in a population of isogenic cells that have been synchronized by serum starvation. Upon growth stimulation, cells reenter the cell cycle from quiescence and undergo the G1/S transition, but not all cells in the population proceed at the same rate. This rate also differs among different cell types [9],[10] and can be modulated by external conditions [11].

To account for the variable transition timing in cell cycle progression, two major types of models have been proposed: the transition probability (TP) models [11]–[15] and the growth-controlled (GC) models [16]–[18]. The TP models attributed temporal variability to random state transitions through different phases of the cell cycle. One of the earliest TP models was proposed to account for the inter-mitotic variability by assuming a single random transition from a non-proliferative A-state to a proliferative B-phase [15]. It was subsequently extended to account for the timing variability in cell cycle reentry starting from quiescent (G0) cells [11],[14]. In this case, the exponential drop in the fraction of G0 cells over time was suggested to indicate a probabilistic nature of the transition. The original model and its subsequent variants have provided excellent fits to various types of experimental data [11]–[15]. However, a major criticism of the TP models is that the transition probability from the A-state was assumed to be time-invariant, despite uneven cell division at mitosis and obvious cell growth or metabolism through the cell cycle [19]. As an alternative, the GC models proposed that the observed temporal variability arises from growth rate heterogeneity within a cell population, rather than random state transitions. Remarkably, this line of models has been able to provide equally good fits to various experimental data [16],[20]. Integrating these two lines of thinking, hybrid models proposed cell-size control and random transitions as regulatory elements for progression to cell division [21],[22]. However, understanding of the underlying mechanisms for cell-size control and random transitions was limited at the time. Consequently, although they provided excellent fits to experimental data, these models remain descriptive to date.

There has been an active debate between these two lines of thinking since their initial propositions. While never fully resolved, the debate gradually faded as the focus in the field of cell cycle studies moved to identifying the dynamical basis for various cell cycle regulations, including the restriction point (R-point) [23], which we have shown to be controlled by a bistable Rb-E2F switch [7]. We also showed that activation of this switch is correlated with the cell's reentry from quiescence into the cell cycle. Interestingly, cell cycle reentry was explored by both the TP and GC models, which were originally developed to describe actively growing cells. For example, the TP models ascribe quiescence and proliferation to low and high transition probabilities, respectively [11],[14]. In addition, the GC models have recently been proposed as an alternative explanation for the “R-point” [18].

**Figure 1:**
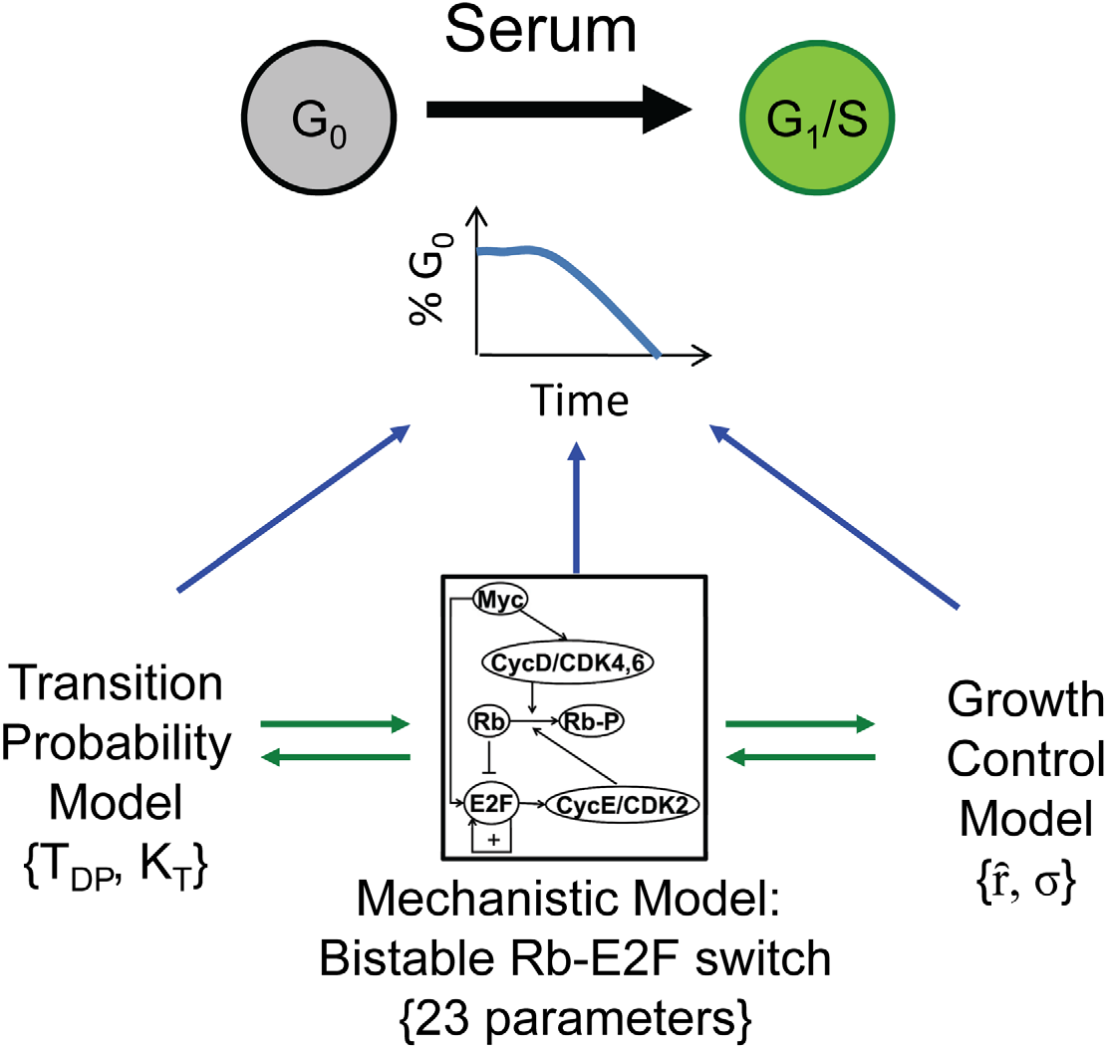
Temporal variability in cell cycle reentry. A population of quiescent cells can undergo the G1/S transition with serum stimulation. The timing of cell cycle entry is highly variable in a cell population, characterized by an exponential drop in the percentage of G0 cells over time (G0 exit curve). To account for such temporal variability, two groups of phenomenological models have been previously proposed: the transition probability (TP) model, which describes the dynamics of cell cycle entry by a transition rate (K_T_) and a time delay of the cell population (T_DP_), and the growth-controlled (GC) model with a mean growth rate (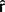) and a variance of the growth rate (σ). We recently demonstrated that the G1/S transition dynamics are governed by a bistable Rb-E2F switch, whose stochastic activation may also account for the G0 exit curve. Here, we propose that the two phenomenological models in essence reflect different aspects of cell cycle reentry dynamics and can be recast into the framework of the mechanistic model.

The temporal variability described by the GC and TP models is based on the distribution of inter-mitotic times and may differ from temporal variability in E2F activation from quiescence. However, we suggest that the stochastic Rb-E2F model embodies the concepts assumed in the TP models and the GC models. Our model predictions and experiments suggest that stochastic activation of E2F can account for temporal variability in cell cycle entry, and the degree of such variability is determined by environmental cues and the regulatory network parameters. These results suggest that the TP and GC models are not mutually exclusive but rather reflect different aspects of the same temporal dynamics in cell cycle entry, as has been speculated [21],[24]. In addition, we show that stochastic activation of the Rb-E2F bistable switch under various environmental conditions can readily be mapped into both TP and GC models with a small number of parameters (Figure 1). We propose that these parameters can potentially serve as concise, quantitative phenotypes of cell states.

## Results

Our recent work has shown that traverse of the R-point is regulated by the Rb-E2F bistable switch [7]. This bistability results from interlocked positive feedback loops embedded in the Myc-Rb-E2F network (Figure S1, see Text S1 for further details). Given the bistable switching property of the Myc-Rb-E2F network, we hypothesized that this network, when subjected to noise, might demonstrate variable timing in E2F activation, which in turn might account for the temporal variability observed in cell cycle entry. This hypothesis is based on the strong correlation we previously observed between E2F activation and DNA synthesis [7]. To test this hypothesis, we developed a stochastic model for the Myc-Rb-E2F network using the chemical Langevin formulation [25],[26] as detailed in Materials and Methods. This formulation allows for implementation of intrinsic and extrinsic noise while retaining the deterministic framework. In this stochastic model, the intrinsic noise arises from the stochastic nature of the biochemical interactions among small numbers of signaling molecules. The extrinsic noise results from heterogeneity in cell size and shape, cell division, or cell cycle stage [27]–[32].

### Modulation of E2F Activation by Serum Stimulation

**Figure 2:**
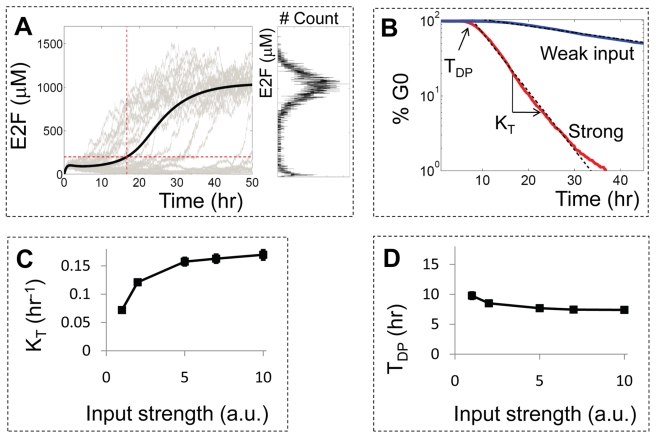
Simulated temporal dynamics in E2F activation using the stochastic Rb-E2F model. (A) Stochastic simulations (25 events) exhibit variable time delays in E2F activation, as shown in gray lines. Two distinct modes of E2F (low and high) are clear and can be separated by a switching threshold (horizontal dotted red line). The inset shows the distribution of E2F at the end of 5,000 simulations (time = 50 h). The minimum time required to reach this threshold is defined as the switching time (vertical dotted red line). In contrast to the stochastic simulations, the deterministic simulation has the same trajectory for a given set of parameters (black line). (B) The percentage of G0 cells over time (G0 exit curve) is plotted for a population of 5,000 simulated cells stimulated at strong (red line, S = 5) and weak (blue line, S = 0.5) input concentrations. The G0 exit curve for the strong input is fitted with an exponential function (black dotted line), 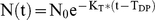; 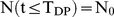, where N_0_ ( = 100%) is the initial percentage of cells in G0, K_T_ is the transition rate, and T_DP_ is the population time delay (see Text S2). For increasing input strength, the transition rate was predicted to increase (K_T_ = 0.029±0.0014 h^−1^ for weak input and 0.16±0.0076 h^−1^ for strong input) and the time delay was predicted to decrease (T_DP_ = 18.0±1.2 h for weak and T_DP_ = 7.7±0.27 h for strong input). (C) K_T_ was predicted to increase with increasing input strength and reach a plateau at sufficiently strong input. The error bars in K_T_ and T_DP_ represent the Monte-Carlo standard deviation of the estimated TP model parameters (see Text S2 for more details). (D) T_DP_ was predicted to decrease with increasing input strength.

The fluctuations in the bistable switch result in significant discrepancies between stochastic and deterministic simulations [33]–[36]. Given a set of initial conditions and parameters in the Myc-Rb-E2F network, the simulated time-courses from a deterministic model are fixed (black line in Figure 2A), but those from a stochastic model show drastically variable trajectories (gray lines in Figure 2A). For example, the stochastic Rb-E2F model can generate two modes of E2F at Time = 50 h when stimulated with weak input as shown in Figure 2A. We define a switching threshold (horizontal red line in Figure 2A) to distinguish the low E2F mode, which corresponds to a non-activated subpopulation of cells, from the high E2F mode, which represents an activated population. This threshold can be used to calculate the percentage of activated cells over time. The minimum time required for E2F to reach the switching threshold is defined as the switching time (vertical red line in Figure 2A, for the deterministic simulation). Similarly, for strong input, the deterministic time-course simulations are fixed and stochastic time-course simulations again show variable trajectories (unpublished data). The distribution of E2F activity in stochastic simulations, however, exhibits a single mode (high E2F level) at strong inputs, rather than two modes as with weak inputs.

Based on our simulations and definitions in Figure 2A, we obtained G0 exit curves for weak and strong input conditions as shown in Figure 2B. These G0 exit curves are analogous to the α-curve in the TP model, which represents the frequency distribution of inter-mitotic times [15]. Both a G0 exit curve and an α-curve can be fitted by an exponential curve with two parameters (black dotted curve in Figure 2B, see Text S2 for further details): transition rate (K_T_) and time delay (T_DP_). This is because both exhibit an initial time delay followed by an exponential drop [11],[14],[15]. The transition rate of the G0 exit curve is inversely proportional to the temporal variability of the cell population. For example, a population of cells with more-synchronous E2F activation would have a higher transition rate than that of a population with less-synchronous E2F activation. If cells were completely synchronized, the G0 exit curve would have an infinite transition rate.

Our simulated E2F activation dynamics predict serum-dependence of both transition rate and time delay. For a weak input (K_T_ = 0.029±0.0014 h^−1^ and T_DP_ = 18.0±1.2 h, blue line in Figure 2B), most cells were expected to remain inactivated and the percentage of G0 cells would decrease slowly. This is because the impact of noise acting on the Rb-E2F bistable switch was only significant enough to activate E2F in some cells, but not in other cells. This would lead to a bimodal distribution of E2F activity (Figure S2A), which is consistent with previous experimental observations in mouse fibroblasts [13],[37],[38]. In contrast, the impact of noise was negligible in the case of strong input and all cells were predicted to be activated at a high transition rate (K_T_ = 0.16±0.0076 h^−1^ and T_DP_ = 7.7±0.27 h, red curve in Figure 2B).The response of the Rb-E2F bistable switch to noise would cause an increase in K_T_ with increasing input strength (Figure 2C) as the population moves from a bimodal to a monomodal distribution at the high mode (Figure S2A). At sufficiently high input strength, further increase in input strength may have a negligible effect on K_T_ (Figure 2C). In contrast, T_DP_ may decrease as the population transitions from a bimodal to a monomodal distribution, and T_DP_ may bottom out at sufficiently high input strength (Figure 2D). The dependence of K_T_ and T_DP_ on input strength can be recapitulated with a minimal bistable model (Figure S2B–D), suggesting that such dependence may be an intrinsic property of bistable systems.

**Figure 3:**
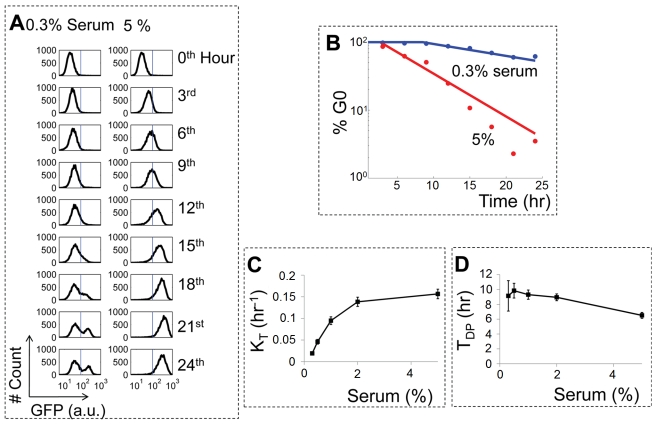
Experimental validation of the predicted temporal dynamics. (A) The temporal dynamics of a cell population depends on serum concentration. At 0th h E2F-d2GFP cells were synchronized in quiescence by serum-starvation (24 h at 0.02% serum). These cells were then stimulated with either 0.3% or 5% serum, and corresponding GFP levels (reporting E2F activity) were determined by flow cytometry. The cell population treated with 0.3% serum exhibited a bimodal distribution of GFP at the 24th h . In contrast, a monomodal distribution was observed at 24th h in the cell population treated with 5% serum. The dotted lines represent switching threshold, which is used to distinguish the low and high modes of E2F. Here, the switching threshold was defined as 2.5 times the variance from the mean of the GFP distribution of serum-starved cells (or GFP distribution at time 0). (B) Serum concentration modulates the temporal dynamics of E2F activation. The thresholds shown as dotted blue lines in (A) were used to calculate the percentage of cells in the low mode of E2F. The two G0 exit curves showed that transition rate increased (K_T_ = 0.031±0.0036 h^−1^ at 0.3% serum and 0.16±0.011 h^−1^ at 5% serum) and time delay decreased (T_DP_ = 5.1±1.1 h at 0.3% and 1.1±0.27 h at 5% serum) with increasing serum concentration. (C) The transition rate increased with serum concentration. (D) The time delay decreased with serum concentration. Data in panels A and B and those in panels C and D were from two independent experiments.

To validate our model predictions, we measured E2F activity in the E2F-d2GFP cell line, which is derived from a rat embryonic fibroblast REF52 cell line and carries a destabilized green fluorescent protein reporter (d2GFP) under the E2F1 promoter. We have shown that this reporter system can be used to monitor E2F activity in response to serum stimulation previously [7]. Prior to serum stimulation, the E2F-d2GFP cells were synchronized at quiescence by serum-starvation (0.02% bovine growth serum, BGS) with basal E2F-GFP expression (Figure 3A). Upon weak serum stimulation (0.3% BGS), only a subpopulation of the cells switched to the high E2F mode over time. At earlier time points (0∼15th h), the difference in E2F level between the non-activated and activated cells was small. The difference between the two modes became increasingly clear, resulting in distinctive bimodality starting at 18th h. In contrast, upon strong serum stimulation (5% BGS), E2F activation was more synchronous. The whole cell population gradually switched to the high mode with greater temporal synchrony without demonstrating detectable bimodality at any tested time point (Figure 3A). It is possible that noise may partition the cell population into two subsets (active and inactive towards proliferation) temporarily even at high serum stimulation. However, simulations suggest that accumulation of E2F in the activated cells at earlier time points may not be significant enough to result in any detectable difference between the two subsets (Figure S2A).

Based on the distribution of E2F in Figure 3A, we calculated the percentage of non-activated cells and obtained a G0 exit curve for each serum condition (Figure 3B). Consistent with predictions in Figure 2B, we observed an increase in K_T_ and decrease in T_DP_ for increasing serum concentration (K_T_ = 0.031±0.0036 h^−1^ and T_DP_ = 5.1±1.1 h at 0.3% serum, and 0.16±0.011 h^−1^ and 1.1±0.27 h at 5% serum), reminiscent of modulation of the α-curve by serum [11],[15]. Consistent with model predictions in Figure 2C–D, we observed increase in K_T_ and decrease T_DP_ for increasing serum concentrations (Figure 3C and 3D). An independent experiment under the same conditions on a different day exhibited similar dependence of K_T_ and T_DP_ on serum (Figure S3).

### Modulation of Stochastic E2F Activation by Strength of CycE-Mediated Feedback

The temporal dynamics of biological systems often depend strongly on network parameters [39].Consequently, the transition rate of cell cycle entry may be modulated by nodal perturbations. This is exemplified in a recent study on the yeast cell cycle [40], which demonstrated that a positive feedback by G1 cyclins is responsible for temporal coherence in gene expression and proper division timing of yeast cells. Loss of this feedback control in the cell cycle machinery was shown to promote incoherent gene expression and abnormal delays of yeast budding. Interestingly, a similar feedback module through a G1 cyclin (CycE) can be found in the Myc-Rb-E2F network also, suggesting its potential role in the control of temporal dynamics.

**Figure 4:**
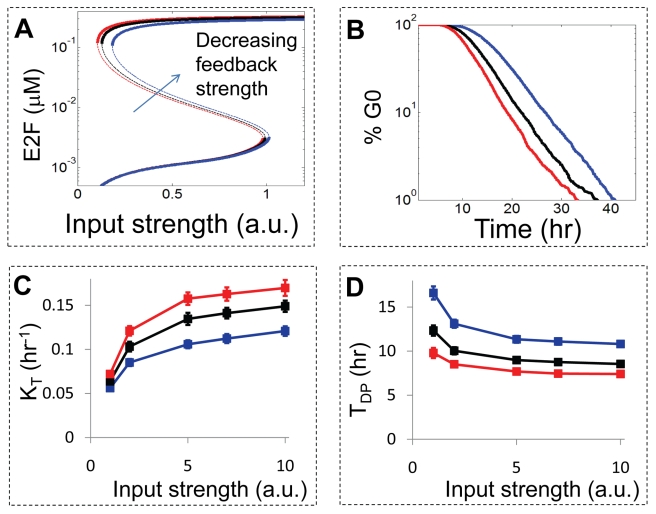
Nodal perturbation at CycE alters temporal dynamics of E2F activation. (A) The strength of the CycE-mediated positive feedback determines the sensitivity of the system to serum stimulation. Bifurcation analyses of the Rb-E2F switch with weak (Rb phosphorylation rate constant k_P4_ = 9 h^−1^, blue), intermediate (k_P4_ = 14 h^−1^, black), and strong strength (k_P4_ = 18 h^−1^, red) of the positive feedback were performed. For decreasing strength of the positive feedback, the system became less sensitive to the input strength, requiring greater critical input strength for E2F activation. (B) The temporal dynamics can be modulated by adjusting the feedback strength. At the saturating input level (S = 10), the Rb-E2F switch was subjected to varying degrees of feedback strength mediated by CycE. G0 exit curves from 5,000 simulations were constructed for strong (red line, *k_P4_* = 18 h^−1^), intermediate (black line, *k_P4_* = 14 h^−1^), and weak (blue line, *k_P4_* = 9 h^−1^) feedback strengths. For decreasing strength of the positive feedback, our simulations predicted a decrease in the transition rate (K_T_ = 0.17±0.0090 h^−1^ for strong, 0.15±0.006 h^−1^ for intermediate, and 00.12±0.0054 h^−1^ for weak feedback strength), and increase the time delay (T_DP_ = 7.4±0.25 h for strong feedback, 8.5±0.53 and 10.8±0.15 h for intermediate and weak feedback strength, respectively). (C) Increase in K_T_ for increasing strength of the positive feedback was predicted for all input strengths. (D) Decrease in T_DP_ for increasing strength of the positive feedback was predicted for all input strengths.

To investigate modulation of the transition rate by nodal perturbations in the Myc-Rb-E2F network, we introduced in silico perturbations of one particular node: the CycE/Cdk2 complex, which forms the CycE-mediated positive feedback loop. Our bifurcation analyses predict that weakening of the CycE-mediated positive feedback loop will desensitize the Rb-E2F bistable switch to serum stimulation, requiring a higher critical serum concentration (Figure 4A) for E2F activation. Similarly, we predict desensitization to serum when CycD is down-regulated or when Rb is up-regulated (unpublished data). Such desensitization is expected to modulate the temporal dynamics of E2F activation. When the positive-feedback strength by CycE is weakened, our simulations in Figure 4B (corresponding simulated distributions in Figure S4) predicted increase in the time delay and decrease in the transition rate. For strong feedback strength, K_T_ was estimated to be 0.17±0.0090 h^−1^. This value was reduced to 0.15±0.006 h^−1^ and 0.12±0.0054 h^−1^ for intermediate and weak feedback strength, respectively. In contrast, T_DP_ for strong feedback input ( = 7.4±0.25 h) was predicted to increase to 8.5±0.53 h for intermediate feedback strength, and to extend further to 10.8±0.15 h for weak feedback strength. Similar dependence of K_T_ and T_DP_ on the feedback strength was predicted for all serum concentrations (Figure 4C and 4D).

**Figure 5:**
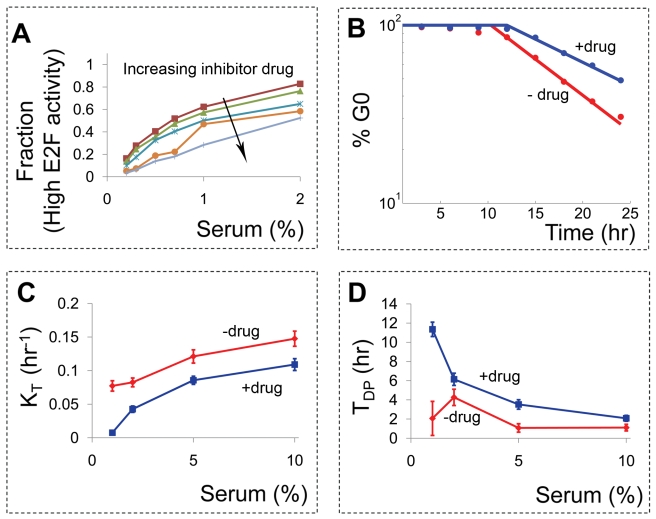
Experimental desensitization of the Rb-E2F switch to serum by a Cdk2 inhibitor drug. (A) E2F activity, measured by the GFP signal in the E2F-d2GFP cells, was assayed under varying concentrations of Cdk2 inhibitor CVT-313 (0.5, 1, 2, 3, and 5 µM) and serum (0.2%, 0.3%, 0.5%, 0.7%, 1.0%, 2.0%). After 24 h of the inhibitor drug treatment in DMEM supplemented with varying serum concentrations, the E2F-d2GFP cells were collected and their GFP signals were assayed by flow cytometry. For each serum and inhibitor drug condition, the fraction of cells with GFP signals above a threshold level was counted and plotted. For a given serum concentration, increasing drug dose led to a decreasing fraction of cells at the high E2F mode. Increasing serum concentration resulted in an increasing fraction of cells at the high E2F mode. (B) The temporal dynamics of E2F activation is altered when CycE-mediated positive feedback is weakened. At 2% serum, we applied Cdk2 inhibitor CVT-313 at 2 µM (blue curve) and monitored the effect on E2F activation over time by flow cytometry. Compared to the case without drug (red curve), the transition rate decreased from 0.078±0.0073 to 0.058±0.0070 h^−1^and the time delay increased from T_DP_ = 9.1±0.70 to T_DP_ = 12.0±0.86 h. (C) Targeting the CycE-mediated positive feedback modulates the transition rate. In an independent set of experiments, time-courses of cell populations treated with varying serum concentrations were obtained for a given drug dose, and the transition rate was calculated for each serum condition. The transition rate increased with serum concentration and reached a plateau at saturating serum concentrations in the absence of the inhibitor, but it continued to increase in the presence of the inhibitor. Overall, K_T_ was greater without the inhibitor than with it. (D) Time delay decreased with increasing serum concentration in the absence of the inhibitor and reached a plateau at the saturating serum concentration. In the presence of the inhibitor, however, T_DP_ continued to decrease. Overall, T_DP_ was greater with the inhibitor than without it.

To test these predictions experimentally, we perturbed the Myc-Rb-E2F network by applying varying concentrations of a cyclin-dependent kinase inhibitor (CVT-313), which has a much higher affinity towards Cdk2 than to other Cdks (Figure S5) [41],[42]. In the context of the current study, which focuses on the cellular dynamics leading to E2F activation, the impact of the Cdk2 inhibitor is primarily the inhibition of the CycE/cdk2 complex. We note that the inhibitor would also affect other components of cell cycle regulation, (e.g., the CycA/cdk2 complex), which were not considered in the model due to their activity mainly downstream of the cell cycle entry point. When the CycE node was perturbed experimentally, we observed inhibitor dose-dependent changes in E2F activity, as measured by GFP fluorescence in the E2F-d2GFP cells. As shown in Figure 5A, increasing dose of the inhibitor drug reduced the fraction of cells in the high E2F mode at 24 h. For example, without the Cdk2 inhibitor, less than 1% serum was required for E2F activation in half of the cell population. With 2 µM Cdk2 inhibitor, 2% serum was required to achieve a similar fraction of E2F activation. Such desensitization to serum stimulation was seen for all inhibitor concentrations tested (Figure 5A).

Next, we tested modulation of temporal dynamics by the Cdk2 inhibitor. At 2% serum, we applied the Cdk2 inhibitor (CVT-313, 2 µM) to monitor its effect on E2F activation over time. Our results in Figure 5B show that the transition rate of the cell population decreased (from K_T_ = 0.078±0.0073 to 0.058±0.0070 h^−1^) and time delay increased (from T_DP_ = 9.1±0.70 to T_DP_ = 12.0±0.86 h) with addition of the Cdk2 inhibitor. Such a decrease in K_T_ with the inhibitor drug is consistent with our model predictions in Figure 4 and was observed for all serum concentrations tested, as shown in Figure 5C (distributions of E2F in Figure S6). As predicted, time delay generally decreased with increasing serum concentrations and it increased in the presence of the Cdk2 inhibitor (Figure 5D). It is noteworthy that the estimated time delay has a large error at low serum concentrations, leading to a non-monotonic dependence of T_DP_ on serum concentrations. This is most likely due to the small number of E2F-activated cells at low serum at earlier time points. This makes estimation of parameters using least squares challenging, giving rise to large errors. We conducted another experiment on a different day under the same experimental conditions and observed similar trends in K_T_ and T_DP_, as shown in Figure S7.

### Mapping Simulated Stochastic E2F Activation into TP and GC Model

Throughout this study, we have analyzed the temporal dynamics of E2F activation by extracting a set of parameters defining the TP model (transition rate and time delay). This parameter extraction establishes a connection with the mechanistic Rb-E2F model. Similarly, the GC model parameters (mean growth rate 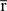 and its variance 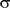, see Text S2 for further details) can be extracted from the stochastic dynamics of E2F activation, and a connection between the GC model and the mechanistic Rb-E2F model can also be established. The GC parameters were estimated from both simulation results in Figure 2 and experimental data in Figure 3, as shown in Table 1. These results show increasing mean growth rate and decreasing variance (normalized to the mean) with increasing input strength.

**Table 1:** Extraction of GC and TP model parameters from simulated and experimentally measured G0 exit curves.

		TP Model Parameters		GC Model Parameters	
	Serum (%)	K_T_ (h^−1^)	T_DP_ (h)	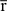 (h^−1^)	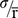 (h^−1^)
Simulations	0.5	0.029±0.0013	18±1.3	0.21±0.034	0.56±0.067
	1	0.073±0.0037	9.7±0.49	0.82±0.12	0.49±0.053
	2	0.12±0.0053	8.5±0.35	2.5±0.31	0.40±0.037
	5	0.16±0.0076	7.7±0.27	4.4±0.72	0.36±0.044
Experiments	0.5	0.050±0.0054	9.8±0.99	0.25±0.19	0.36±0.067
	1	0.09±0.0089	9.3±0.62	1.9±0.45	0.80±0.13
	2	0.14±0.011	8.9±0.46	4.1±0.92	1.4±0.24
	5	0.16±0.011	6.5±0.40	3.2±0.70	1.3±0.21

In both simulations and experiments, we varied serum concentration only, while keeping all else the same. For each G0 exit curve, we extracted the TP and GC model parameters.

**Figure 6:**
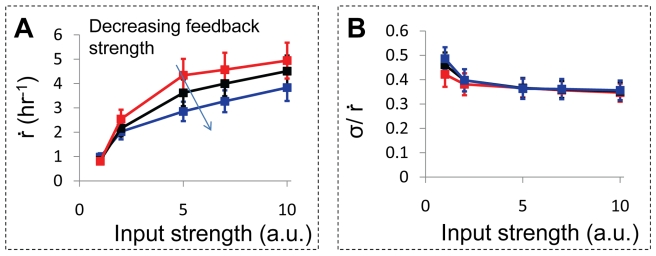
Mapping the stochastic dynamics of E2F activation with the GC model. Simulation results from the stochastic Rb-E2F model are fitted to the GC model with two parameters (adapted from the G1-rate model [16]), which is defined as 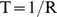, where 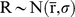. R is a random variable normally distributed with the mean growth rate 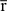 over the entire cell population and σ is the standard deviation of the growth rate. The two parameters of the GC model were approximated using least squares, and their error bars represent the Monte-Carlo standard deviations (see Text S2). (A) Our simulations predicted increasing growth rate for increasing input strengths and positive feedback strengths (k_P4_ = 9, 14, and 18 h^−1^ for blue, black, and red lines, respectively). (B) No significant change in the normalized variance was predicted.

In addition, we predicted the dependence of the strength of the CycE-mediated positive feedback on the GC model parameters, as shown in Figure 6. Consistent with Table 1, our results predicted increasing growth rate (Figure 6A) and decreasing normalized variance (Figure 6B) for increasing input strength. However, decreasing the strength of the CycE-mediated positive feedback was predicted to reduce mean growth rate without affecting its normalized variance significantly. Such parameter extraction defining the phenomenological models provides a quantitative mapping between the phenomenological models and the mechanistic Rb-E2F model. It is noteworthy that both TP and GC models fit the data with comparable levels of uncertainty in the estimated parameters, suggesting that both models may provide similarly good fits to the stochastic dynamics of E2F activation.

## Discussion

Focusing on E2F activation, we have shown that the temporal variability in cell cycle entry from quiescence can be quantitatively modeled by stochastic activation of a bistable Rb-E2F switch [7]. In addition, we have shown that the degree of such variability can be modulated by varying the input strength or by perturbing the network parameters.

Our model predictions are overall consistent with experimental measurements. In particular, our analysis indicates that serum and a Cdk2 inhibitor drug exert opposite influences on the temporal dynamics of E2F activation: transition rate increases and time delay decreases with increasing serum, but transition rate decreases and time delay increases with increasing Cdk2 inhibitor concentrations. We suggest that such a well-calibrated stochastic model for the Rb-E2F switch may guide further experimental analyses to gain insights into the system-level dynamics underlying cell cycle entry. For example, our model predicts that reducing the CycD/Cdk4,6 activity may have similar effects on temporal dynamics of E2F activation as the Cdk2 inhibitor, while knocking down Rb may increase transition rate (unpublished data). In addition, we can predict stochastic dynamics of E2F activation under combinatorial perturbations including growth factors, inhibitor drugs targeting the Myc-Rb-E2F network, or mutations within this network.

Throughout this study, we have focused on a single transition during cell cycle progression (quiescence to proliferation) due to its experimental and computational tractability. To further simplify analysis, we have chosen not to model cell division or growth explicitly. Instead, the variability associated with these processes is lumped into the extrinsic noise terms in our SDE model. More explicit mechanisms to account for such variability may further improve the quantitative agreement between the modeling and the experiment. For example, our simulation results suggest that the major source of noise is extrinsic noise, while variability in the initial conditions can lead to minor yet discernable change in the temporal dynamics of E2F activation. This is evident when E2F activation dynamics are compared under two conditions: varying initial conditions and varying variance of the extrinsic noise (ω) in the stochastic model (see Materials and Methods). At a fixed variance of the extrinsic noise, increasing variability in the initial conditions (Gaussian-distributed with the mean being the base initial conditions and various variance values, Var) is predicted to decrease transition rate and time delay (Figure S8A–B). Similarly, increasing ω without any variability in the initial conditions (Var = 0) is predicted to decrease transition rate and time delay (Figure S8C–D), but these changes by extrinsic noise are predicted to be significantly greater than those by initial conditions. These decreases in K_T_ and increases in T_DP_ reflect the loss of synchrony in E2F activation due to increasing extrinsic noise or initial condition variability. This may explain reduced time delay in actively growing cells compared to that in quiescent cells [14].

Equally important, we further show that these predicted stochastic dynamics of the Rb-E2F model can be quantitatively mapped into two lines of phenomenological models reflecting seemingly conflicting views: the TP model and the GC model. For a given set of parameters defining the stochastic model, the simulated stochastic E2F activation at the population level can be uniquely described by a set of parameters defining the TP model or the GC model (compare Figure 4C–D and Figure 6A–B). Furthermore, different sets of parameters in the stochastic model would lead to different parameters in the TP or the GC models. We propose that this mapping provides a simple conceptual framework that reconciles the different views reflected in the TP and GC models, which have been a source of unresolved debate over the last several decades. In other words, the stochastic model can be considered as a common mechanistic basis for the two seemingly different models.

During the mapping from our stochastic model to the TP or GC models, details associated with individual signaling reactions are necessarily lost in the resulting TP or GC models, pointing to their limitations in offering mechanistic insights. However, a by-product of this mapping is a potential, unappreciated utility of the TP and GC models. On one hand, these phenomenological models are simple and are able to provide quantitative description of the population-level dynamics associated with variable cell cycle entry. On the other, specific changes in the underlying reaction networks can be manifested in changes in the parameters in these simple models. As such, together with a mechanistically based model, the TP and GC models can serve as a concise platform to define quantitative phenotypes that facilitate classification of cell types or cell states.

This utility may be particularly useful for cancer diagnosis, since most cancers have defects in the Myc-Rb-E2F signaling pathway [43],[44]. Recent approaches for cancer classification involve microarray-based gene expression profiling to develop cancer signatures [45], which have been used to reveal the activation status of oncogenic signaling pathways [46]. Here we suggest that oncogenic phenotypes resulting from deregulation in these pathways may also serve as cancer signatures. Using the mapping technique defined in this work, we can develop a library of predicted phenotypes (defined as TP or GC model parameters) based on the Myc-Rb-E2F network under various nodal mutations or stimulatory inputs. This library can be correlated with the oncogenic phenotypes (defined as TP or GC model parameters) of an unknown cancer cell type. In principle, this correlation can be used to infer the activation status of the Myc-Rb-E2F network of the cancer cell type. For a small number of test conditions, this may be challenging owing to the stochastic dynamics of cell cycle entry. However, increasing the number of test conditions may enhance the diagnostic potential of this approach.

## Materials and Methods

### Development of a Stochastic Rb-E2F Model

The deterministic version of the Rb-E2F model, developed in our previous work [7], served as a basis for the stochastic Rb-E2F model. To capture stochastic aspects of the Rb-E2F signaling pathway, we adopt the chemical Langevin formulation [25],[26],[47] as shown in Eqn (1).
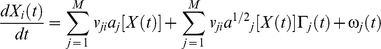
where *X_i_(t)* represents the number of molecules of a molecular species *i* (*i* = 1, …, *N*) at time *t*, and *X(t)* = (*X_1_(t)*, …, *X_N_(t)*) is the state of the entire system at time *t*. *X(t)* evolves over time at the rate of *a_j_*[*X(t)*] (*j* = *1*, …, *M*), and the corresponding change in the number of individual molecules is described in *v_ji_*. 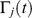 and 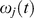 are temporally uncorrelated, statistically independent Gaussian noises. This formulation retains the deterministic framework (the first term), and reaction-dependent and reaction-independent noise. The concentration units in the deterministic model were converted to molecule numbers, so that the mean molecule number for E2F would be approximately 1,000. We assumed a mean of 0 and variance of 1 for Γ*_j_* (*t*), and a mean of 0 and appropriately determined variance for *ω_j_* (*t*) (see Text S1 for more details). The resulting stochastic differential equations (SDEs) were implemented and solved in Matlab.

### Cell Culture Conditions

Actively growing E2F-d2GFP cells [7] were serum-starved in Dulbecco's modified Eagle's medium (DMEM) with 0.02% of bovine growth serum (BGS). After 24 h of serum starvation, they were stimulated with varying serum concentrations for cell cycle entry in the presence or absence of Cdk2 inhibitor CVT-313 (from Calbiochem: Cat #238803). Cell cycle progression was blocked at the DNA synthesis stage by hydroxyurea (HU block), which we have shown has insignificant impact on the GFP signal [7]. At various time points, these cells were collected and fixed in 1% formaldehyde for fluorescence assay.

### Fluorescence Assay with Flow Cytometry

E2F-d2GFP rat embryonic fibroblasts were assayed for a destabilized green fluorescent protein reporter (d2GFP) for E2F activity. The intensity of d2GFP was measured with a flow cytometry system (BD FACSCanto II).

### Western Blots

E2F-d2GFP cells were serum-starved (BGS = 0.02%) for 24 h before they were treated with varying concentration of the Cdk2 inhibitor (CVT-313, EMD # 238803) and serum. After 24 h of serum/inhibitor drug treatment, cell lysates were collected and Western blotting was conducted with primary antibodies recognizing Rb phosphorylation at Cdk4-specific serine 780 (Santa Cruz, #sc-12901-R) and at Cdk2-specific threonine 821 (Santa Cruz, #sc-16669-R). These were conjugated with anti-rabbit secondary antibodies (GE Healthcare, #NA934) for detection. As a loading control, actin was measured with actin-recognizing primary antibodies (Santa Cruz, #sc-8432) conjugated with anti-mouse secondary antibodies (GE Healthcare, #NA9310).

## Supporting Information

Figure S1 **A schematic of the Rb-E2F bistable switch.** Here, Rb represents the entire Rb family (pRB, p107, and p130) and E2F represents all activating E2Fs (E2F1, E2F2, and E2F3a). In quiescent cells E2F is bound by Rb and its transcriptional activities are repressed. Growth stimulation removes the Rb repression by upregulating cyclin D (CycD), which, in complex with Cdk4,6, phosphorylates Rb to release E2F. In addition, growth stimulation induces a transcription factor Myc that upregulates CycD. The free form of E2F synergizes with Myc to induce its own transcription, forming feed-forward and positive feedback loops. Subsequently, E2F activates the transcription of Cyclin E (CycE), which forms a complex with Cdk2 to further remove Rb repression by phosphorylation, constituting another positive feedback loop.
(0.39 MB TIF)

Figure S2
**Simulated temporal dynamics of E2F activation by the full model and a minimal model.** (A) The Rb-E2F bistable switch was stimulated with weak (S = 0.5) and strong (S = 5) input strengths. E2F distributions from 1,000 simulations were sampled at various time points for both conditions. For weak input strength, bimodality was predicted to emerge at around 16th hour. At strong input strength, however, bimodality was expected to be less clear. (B) A minimal model can be used to recapitulate the temporal dynamics of the bistable Rb-E2F switch. The model describes activity of a molecule X: 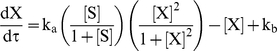, where S is the input strength, k_a_ ( = 5) is the lumped rate term for synthesis and feedback strength, and k_b_ ( = 0.1) is a basal synthesis term. Bifurcation analysis of the minimal bistable model shows hysteresis. (C) This minimal model was converted to a stochastic model using the chemical Langevin formulation. The transition rates were calculated for cell populations stimulated at various input strengths. The transition rate increased with input strength and reached a plateau at sufficiently high input strength. (D) In the minimal bistable model, the time delay decreased with increasing input strength and reached a plateau at sufficiently high input strength.(0.71 MB TIF)

Figure S3 **Independent time-course measurements of GFP signal reporting activity.** GFP signal under the same experimental conditions as Figure 3 was measured at varying time points with flow cytometry. (A) For 0.3% serum, the transition rate and time delay were estimated to be 0.022±0.0041 h^−1^ and 10.0±1.8 h, respectively. At high serum concentration ( = 5%), the transition rate increased to 0.11±0.0099 h^−1^ and time delay decreased to 7.9±0.55 h. (B) K_T_ increased with serum concentration. (C) T_DP_ decreased with serum concentration.
(0.28 MB TIF)

Figure S4 **Predicted modulation of the temporal dynamics of E2F activation.** Temporal dynamics of E2F activation were simulated at varying input strengths (weak→S = 0.5, intermediate→S = 1, and strong→S = 5) and varying CycE-mediated positive feedback strengths (strong→k_P4_ = 18 h^−1^ and weak→k_P4_ = 9 h^−1^). With strong positive feedback (PFB), bimodality was predicted for weak input while monomodality (E2F ON) was predicted for intermediate and strong stimulations. With weak positive feedback, the percentage of E2F activation was predicted to decrease for weak and intermediate input strengths. For strong input, however, the effect of the positive feedback strength was minor.
(0.54 MB TIF)

Figure S5 **Specificity of the Cdk2 inhibitor.** To demonstrate the effect of the Cdk2 inhibitor on Cdk2 kinase activity, we measured Rb phosphorylation at the Cdk2-specific and Cdk4-specific residues for varying inhibitor concentrations. An isogenic population of serum-starved E2F-d2GFP cells was used for Western blotting. In serum-starvation condition (serum = 0.02%), Rb phosphorylation at either residue was negligible. With serum stimulation (serum = 10%), a significant increase in Rb phosphorylation at both residues was observed. For increasing Cdk2 inhibitor concentration, Rb phosphorylation efficiency decreased at the Cdk2-specific residue, but no significant change was observed at the Cdk4-specific residue.
(0.48 MB TIF)

Figure S6 **Experimentally measured E2F time courses for varying serum concentrations, in the absence or presence of the Cdk2 inhibitor drug (at 2 µM).** At 0th h E2F-d2GFP cells were synchronized in quiescence by serum-starvation (24 h at 0.02% serum), stimulated with varying serum concentrations (with or without the Cdk2 inhibitor drug), and measured for GFP (reporting E2F activity) by flow cytometry at the indicated time points.
(1.62 MB TIF)

Figure S7 **E2F time-courses under varying serum concentrations in the absence or presence of Cdk2 inhibitor (at 2 µM) performed on a separate day.** (A) G0 exit curves in the absence and presence of the Cdk inhibitor (2% BGS). Addition of the inhibitor reduced the transition rate from 0.10±0.0081 to 0.090±0.0091 h^−1^ and increased the time delay from 7.7±0.55 to 9.1±0.67 h. (B) Transition rate as a function of serum concentration in the absence or presence of the Cdk2 inhibitor. (C) Time delay as a function of serum concentration in the absence or presence of the Cdk2 inhibitor. (D) E2F distribution over time in the presence or absence of the Cdk2 inhibitor.
(0.80 MB TIF)

Figure S8 **Variability in the initial conditions versus in the rates of the chemical reactions.** The effects of variability in the initial conditions and in the rates of the chemical reactions were evaluated on the temporal dynamics of E2F activation. With all else the same, our simulation results predicted that transition rate (A) and time delay (B) would decrease significantly as ω was increased from 25 to 50. To describe variability in the initial condition, we assumed that the initial concentrations for Rb and the Rb-E2F complex were Gaussian-distributed with the mean being their base value and varying variance levels. At a fixed variance of extrinsic noise (ω = 50), our simulation results predicted that transition rate (C) and time delay (D) would decrease slightly with increasing variance of the initial conditions. Overall, the activation dynamics of E2F is much more sensitive to changes in extrinsic variability than those in the initial condition.
(0.25 MB TIF)

Text S1 **Model development.**
(0.27 MB DOC)

Text S2 **Calculation of metrics: TP and GC model parameters.**
(0.04 MB DOC)

## Abbreviations

GC: growth control
TP: transition probability
