# Supplementary figures and images for: Stochastic E2F Activation and Reconciliation of Phenomenological Cell-Cycle Models
Source: PLoS Biol. 2010 Sep 21;8(9):e1000488. doi: 10.1371/journal.pbio.1000488 (PMC2943438; doi:10.1371/journal.pbio.1000488)

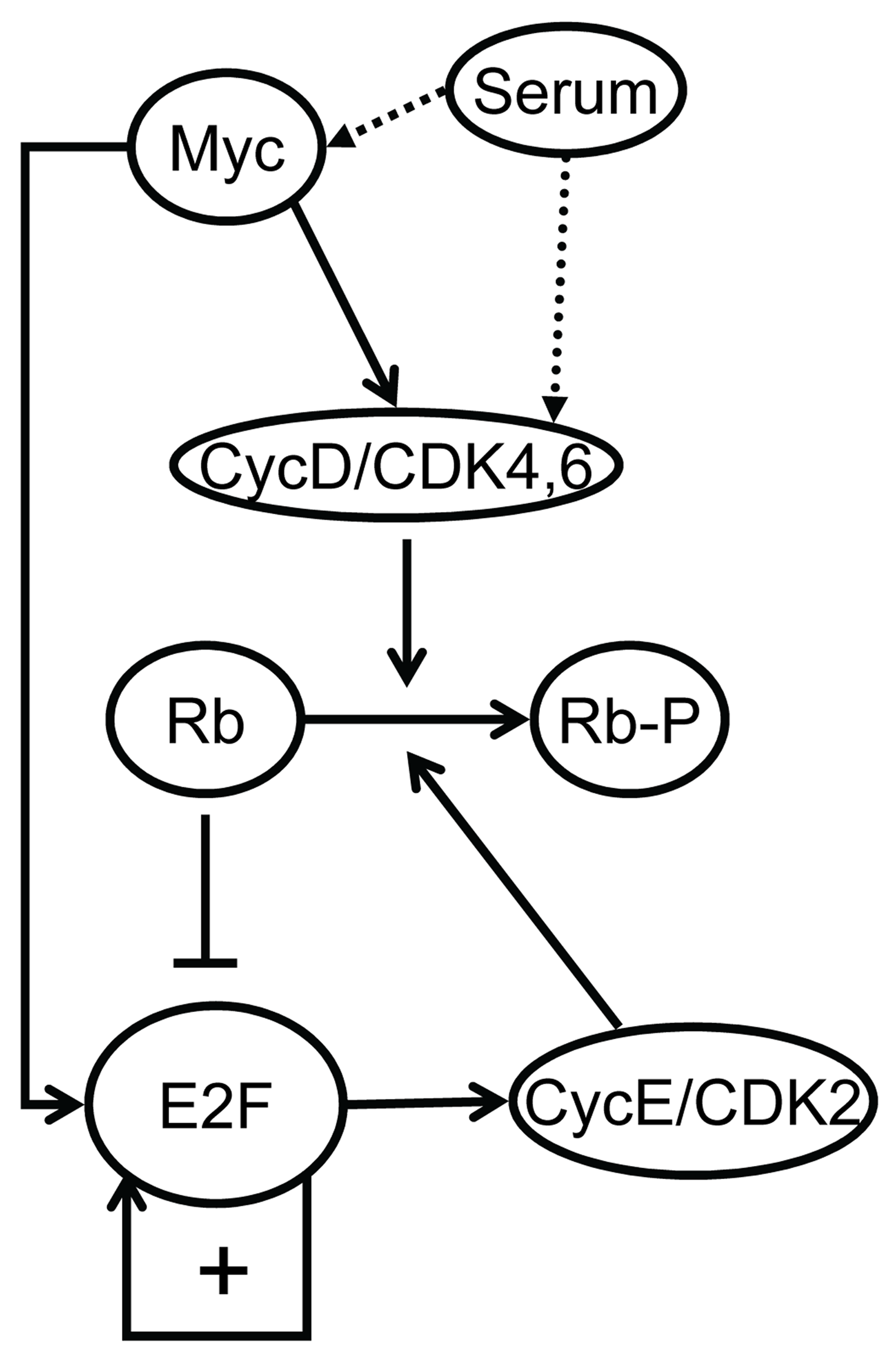

Supplement: Figure S1 — A schematic of the Rb-E2F bistable switch. Here, Rb represents the entire Rb family (pRB, p107, and p130) and E2F represents all activating E2Fs (E2F1, E2F2, and E2F3a). In quiescent cells E2F is bound by Rb and its transcriptional activities are repressed. Growth stimulation removes the Rb repression by upregulating cyclin D (CycD), which, in complex with Cdk4,6, phosphorylates Rb to release E2F. In addition, growth stimulation induces a transcription factor Myc that upregulates CycD. The free form of E2F synergizes with Myc to induce its own transcription, forming feed-forward and positive feedback loops. Subsequently, E2F activates the transcription of Cyclin E (CycE), which forms a complex with Cdk2 to further remove Rb repression by phosphorylation, constituting another positive feedback loop. (0.39 MB TIF) [file pbio.1000488.s001.tif]

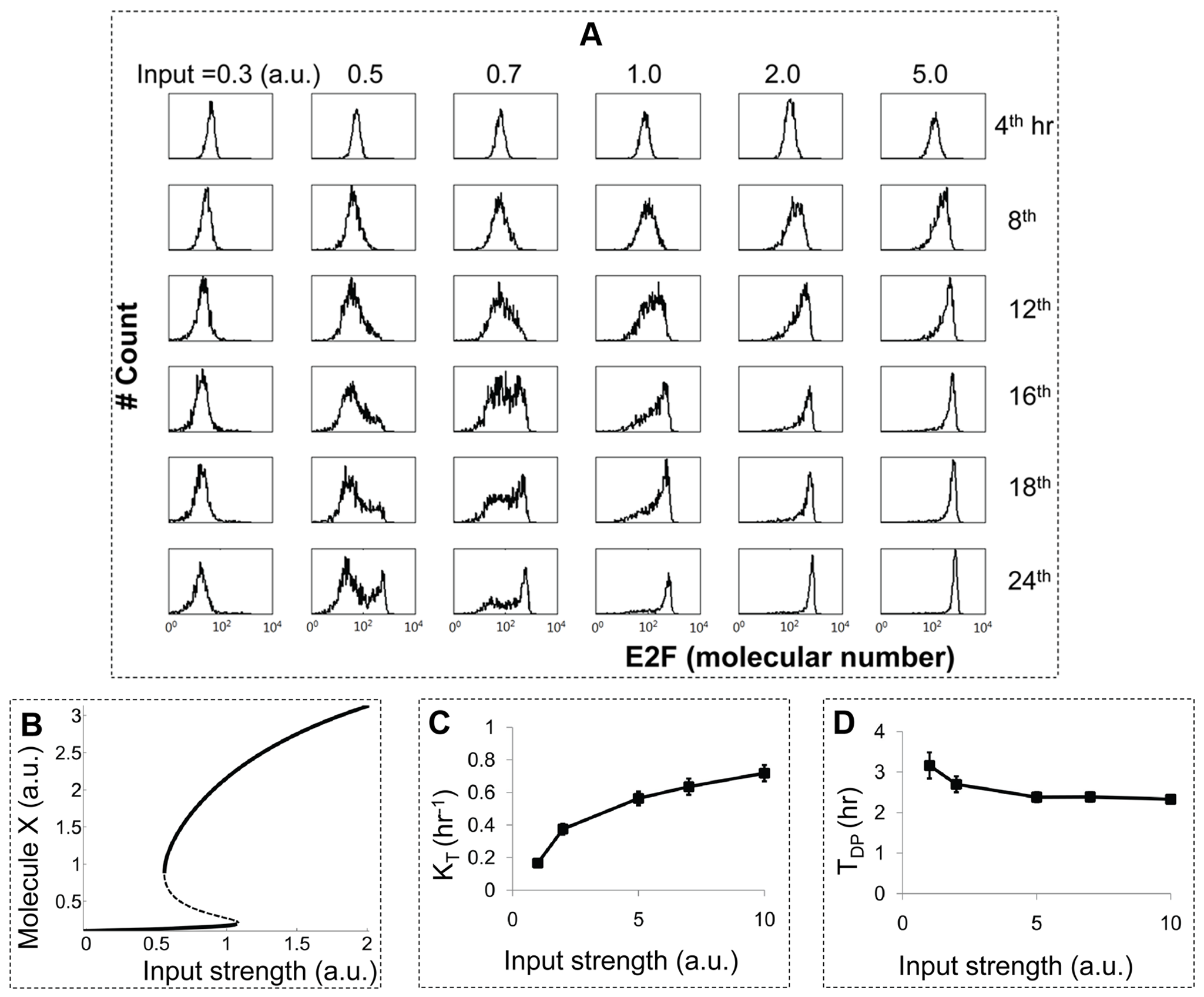

Supplement: Figure S2 — Simulated temporal dynamics of E2F activation by the full model and a minimal model. (A) The Rb-E2F bistable switch was stimulated with weak (S = 0.5) and strong (S = 5) input strengths. E2F distributions from 1,000 simulations were sampled at various time points for both conditions. For weak input strength, bimodality was predicted to emerge at around 16th hour. At strong input strength, however, bimodality was expected to be less clear. (B) A minimal model can be used to recapitulate the temporal dynamics of the bistable Rb-E2F switch. The model describes activity of a molecule X: , where S is the input strength, ka ( = 5) is the lumped rate term for synthesis and feedback strength, and kb ( = 0.1) is a basal synthesis term. Bifurcation analysis of the minimal bistable model shows hysteresis. (C) This minimal model was converted to a stochastic model using the chemical Langevin formulation. The transition rates were calculated for cell populations stimulated at various input strengths. The transition rate increased with input strength and reached a plateau at sufficiently high input strength. (D) In the minimal bistable model, the time delay decreased with increasing input strength and reached a plateau at sufficiently high input strength. (0.71 MB TIF) [file pbio.1000488.s002.tif]

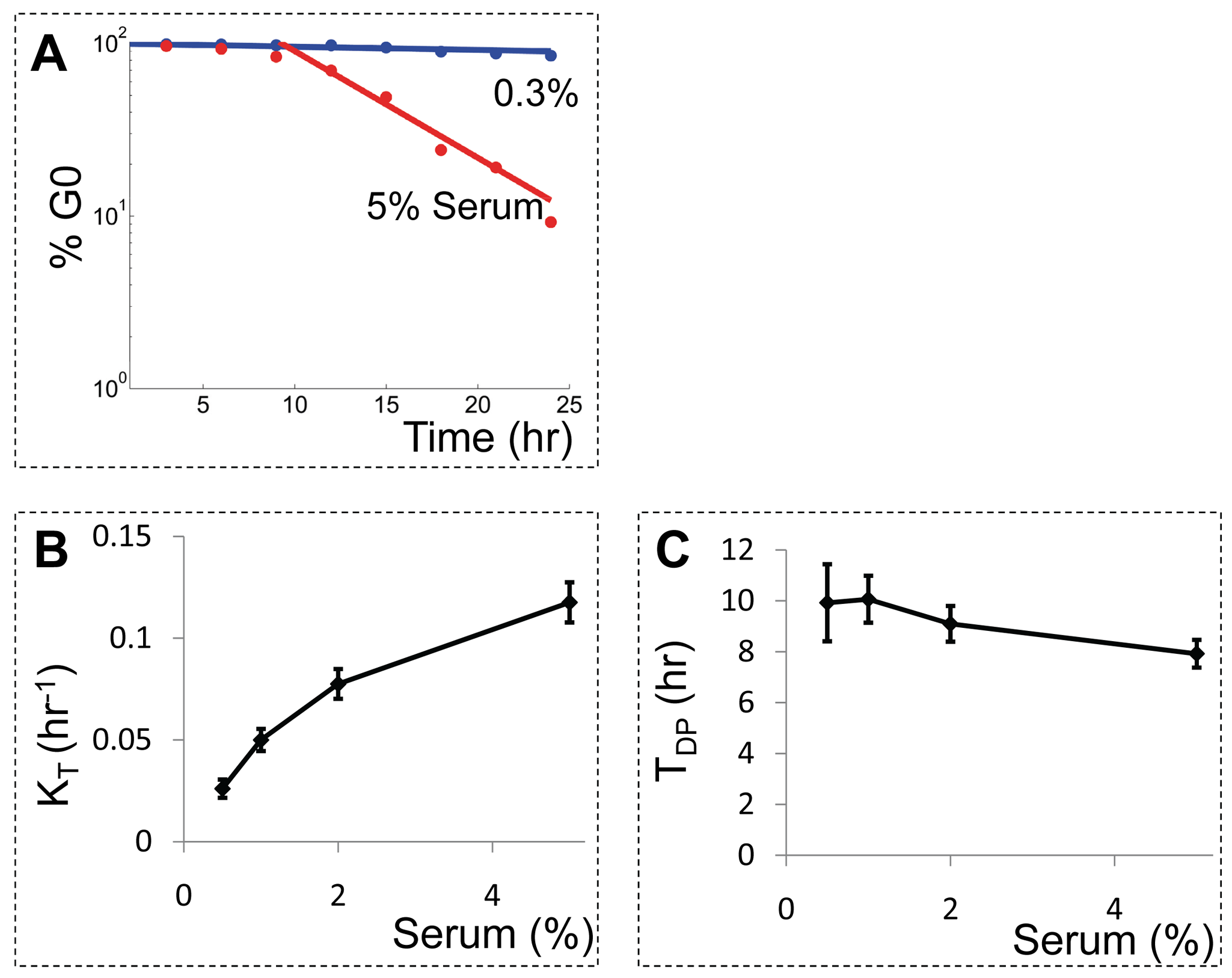

Supplement: Figure S3 — Independent time-course measurements of GFP signal reporting activity. GFP signal under the same experimental conditions as Figure 3 was measured at varying time points with flow cytometry. (A) For 0.3% serum, the transition rate and time delay were estimated to be 0.022±0.0041 h−1 and 10.0±1.8 h, respectively. At high serum concentration ( = 5%), the transition rate increased to 0.11±0.0099 h−1 and time delay decreased to 7.9±0.55 h. (B) KT increased with serum concentration. (C) TDP decreased with serum concentration. (0.28 MB TIF) [file pbio.1000488.s003.tif]

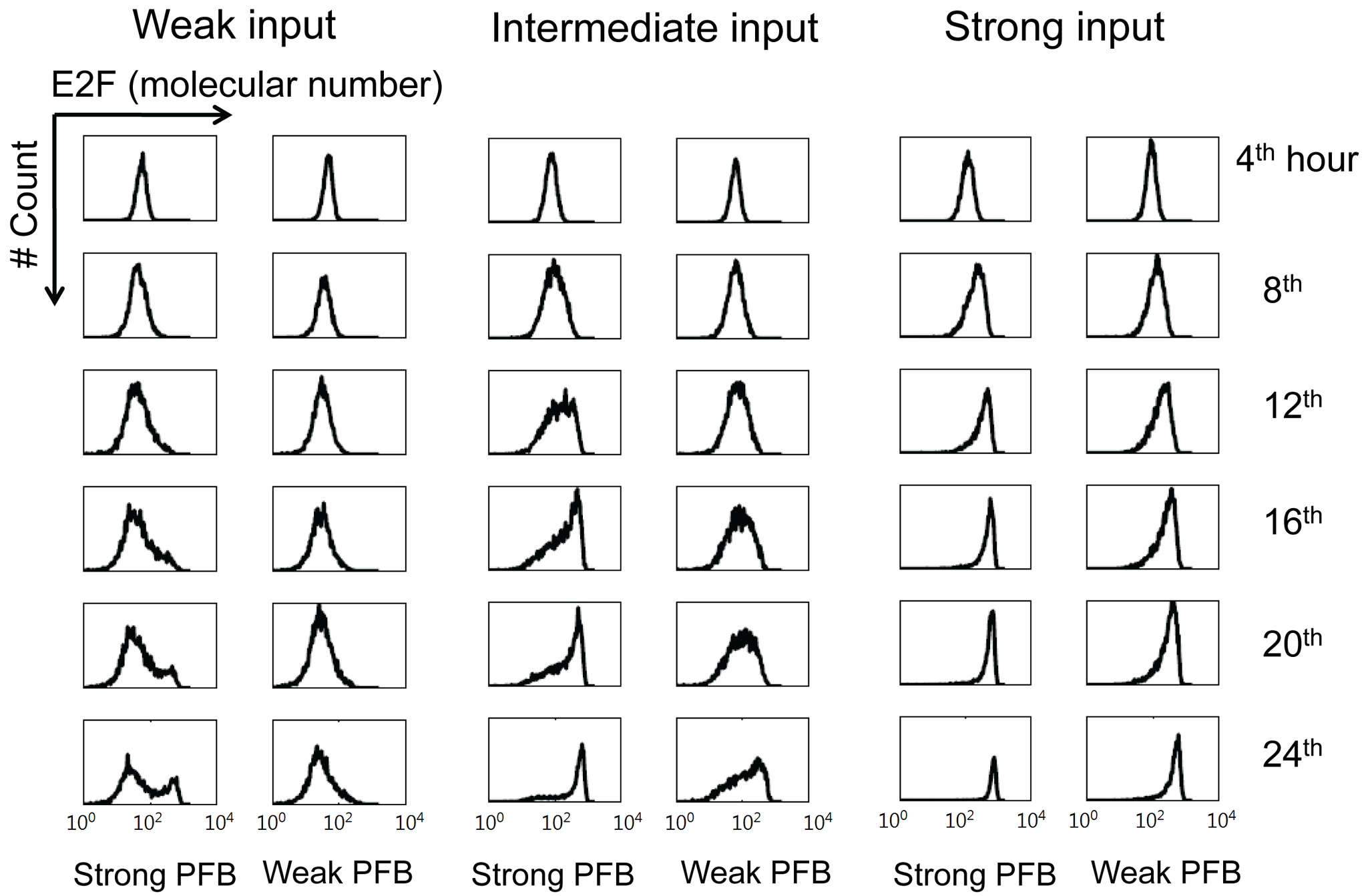

Supplement: Figure S4 — Predicted modulation of the temporal dynamics of E2F activation. Temporal dynamics of E2F activation were simulated at varying input strengths (weak→S = 0.5, intermediate→S = 1, and strong→S = 5) and varying CycE-mediated positive feedback strengths (strong→kP4 = 18 h−1 and weak→kP4 = 9 h−1). With strong positive feedback (PFB), bimodality was predicted for weak input while monomodality (E2F ON) was predicted for intermediate and strong stimulations. With weak positive feedback, the percentage of E2F activation was predicted to decrease for weak and intermediate input strengths. For strong input, however, the effect of the positive feedback strength was minor. (0.54 MB TIF) [file pbio.1000488.s004.tif]

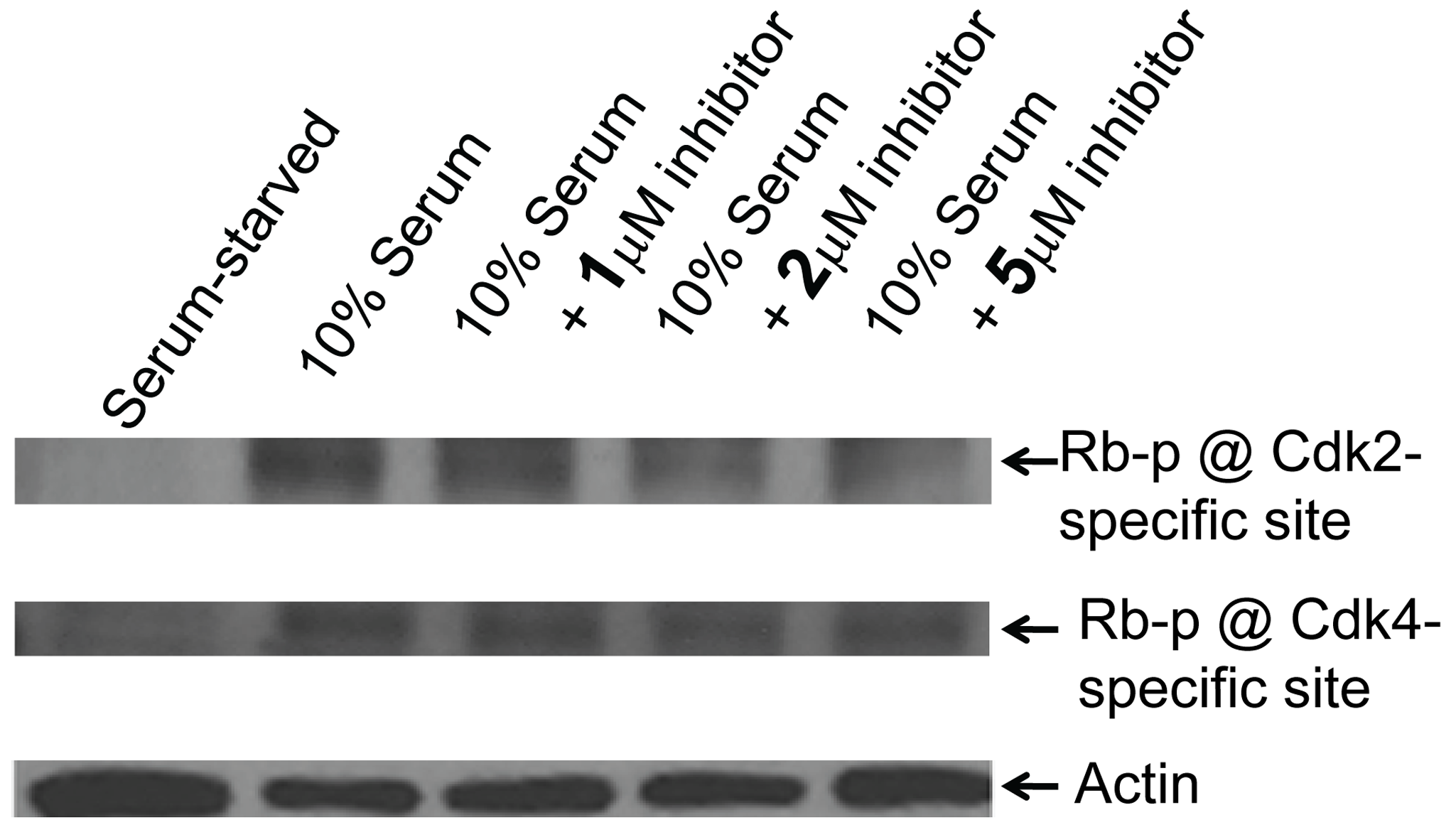

Supplement: Figure S5 — Specificity of the Cdk2 inhibitor. To demonstrate the effect of the Cdk2 inhibitor on Cdk2 kinase activity, we measured Rb phosphorylation at the Cdk2-specific and Cdk4-specific residues for varying inhibitor concentrations. An isogenic population of serum-starved E2F-d2GFP cells was used for Western blotting. In serum-starvation condition (serum = 0.02%), Rb phosphorylation at either residue was negligible. With serum stimulation (serum = 10%), a significant increase in Rb phosphorylation at both residues was observed. For increasing Cdk2 inhibitor concentration, Rb phosphorylation efficiency decreased at the Cdk2-specific residue, but no significant change was observed at the Cdk4-specific residue. (0.48 MB TIF) [file pbio.1000488.s005.tif]

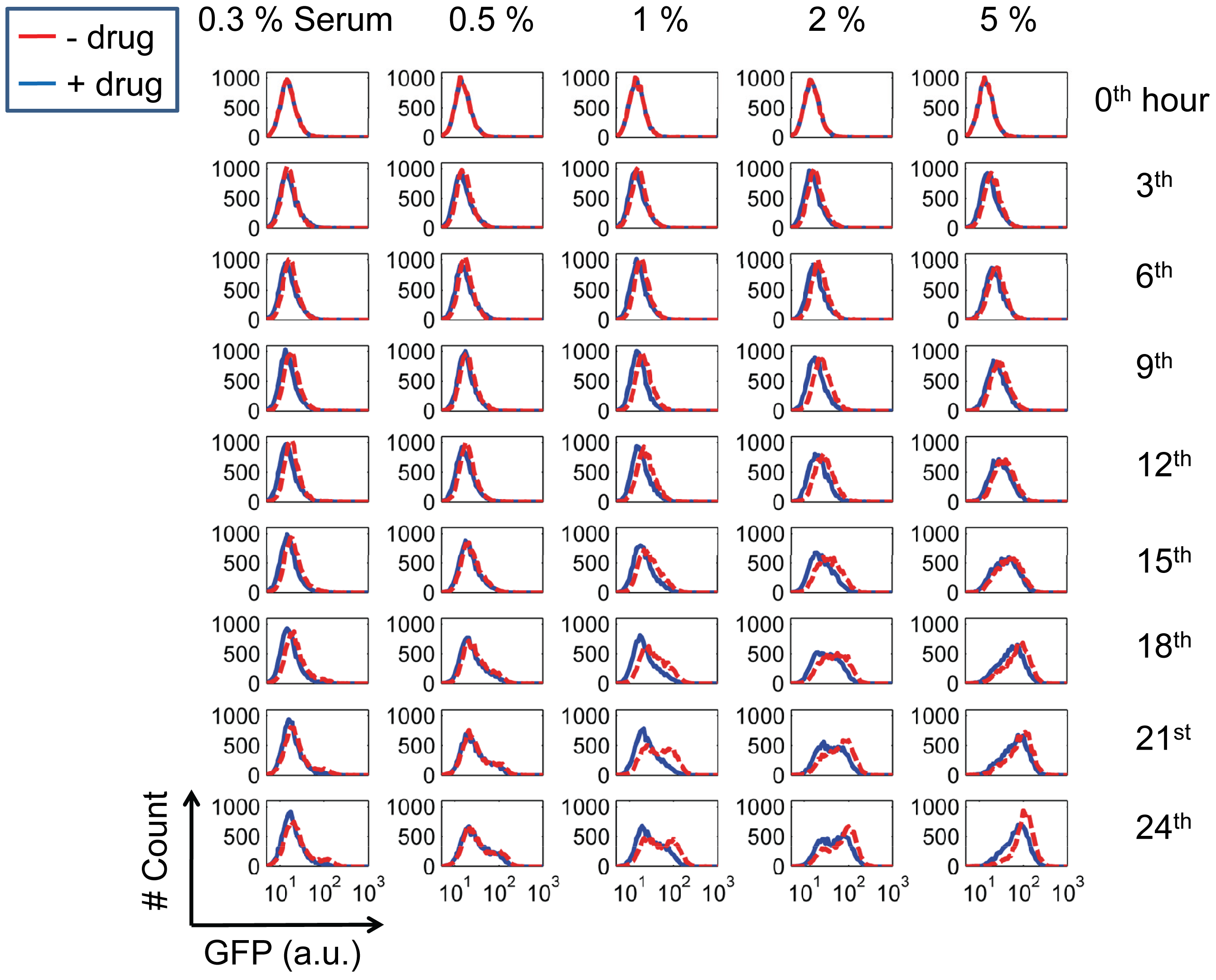

Supplement: Figure S6 — Experimentally measured E2F time courses for varying serum concentrations, in the absence or presence of the Cdk2 inhibitor drug (at 2 µM). At 0th h E2F-d2GFP cells were synchronized in quiescence by serum-starvation (24 h at 0.02% serum), stimulated with varying serum concentrations (with or without the Cdk2 inhibitor drug), and measured for GFP (reporting E2F activity) by flow cytometry at the indicated time points. (1.62 MB TIF) [file pbio.1000488.s006.tif]

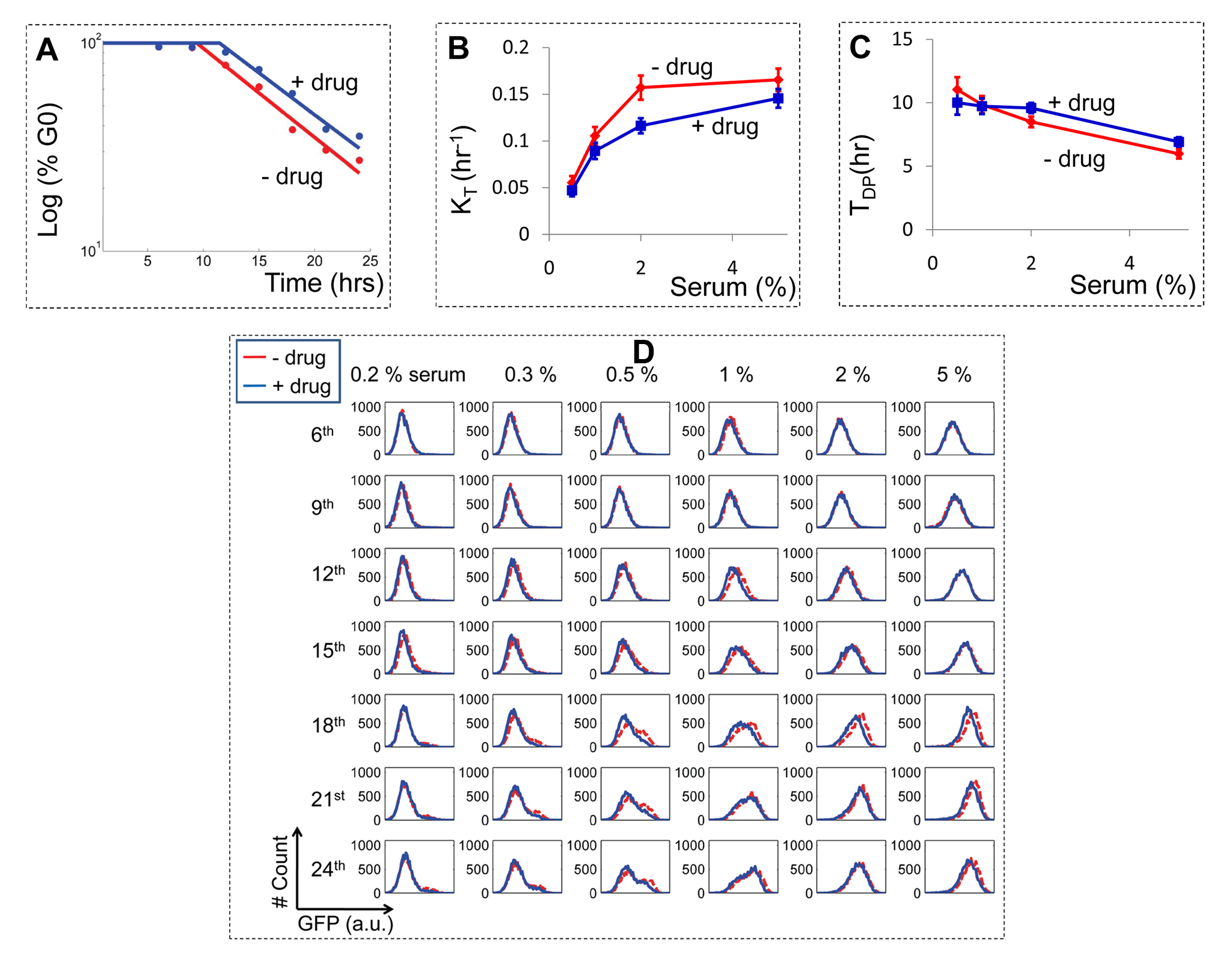

Supplement: Figure S7 — E2F time-courses under varying serum concentrations in the absence or presence of Cdk2 inhibitor (at 2 µM) performed on a separate day. (A) G0 exit curves in the absence and presence of the Cdk inhibitor (2% BGS). Addition of the inhibitor reduced the transition rate from 0.10±0.0081 to 0.090±0.0091 h−1 and increased the time delay from 7.7±0.55 to 9.1±0.67 h. (B) Transition rate as a function of serum concentration in the absence or presence of the Cdk2 inhibitor. (C) Time delay as a function of serum concentration in the absence or presence of the Cdk2 inhibitor. (D) E2F distribution over time in the presence or absence of the Cdk2 inhibitor. (0.80 MB TIF) [file pbio.1000488.s007.tif]

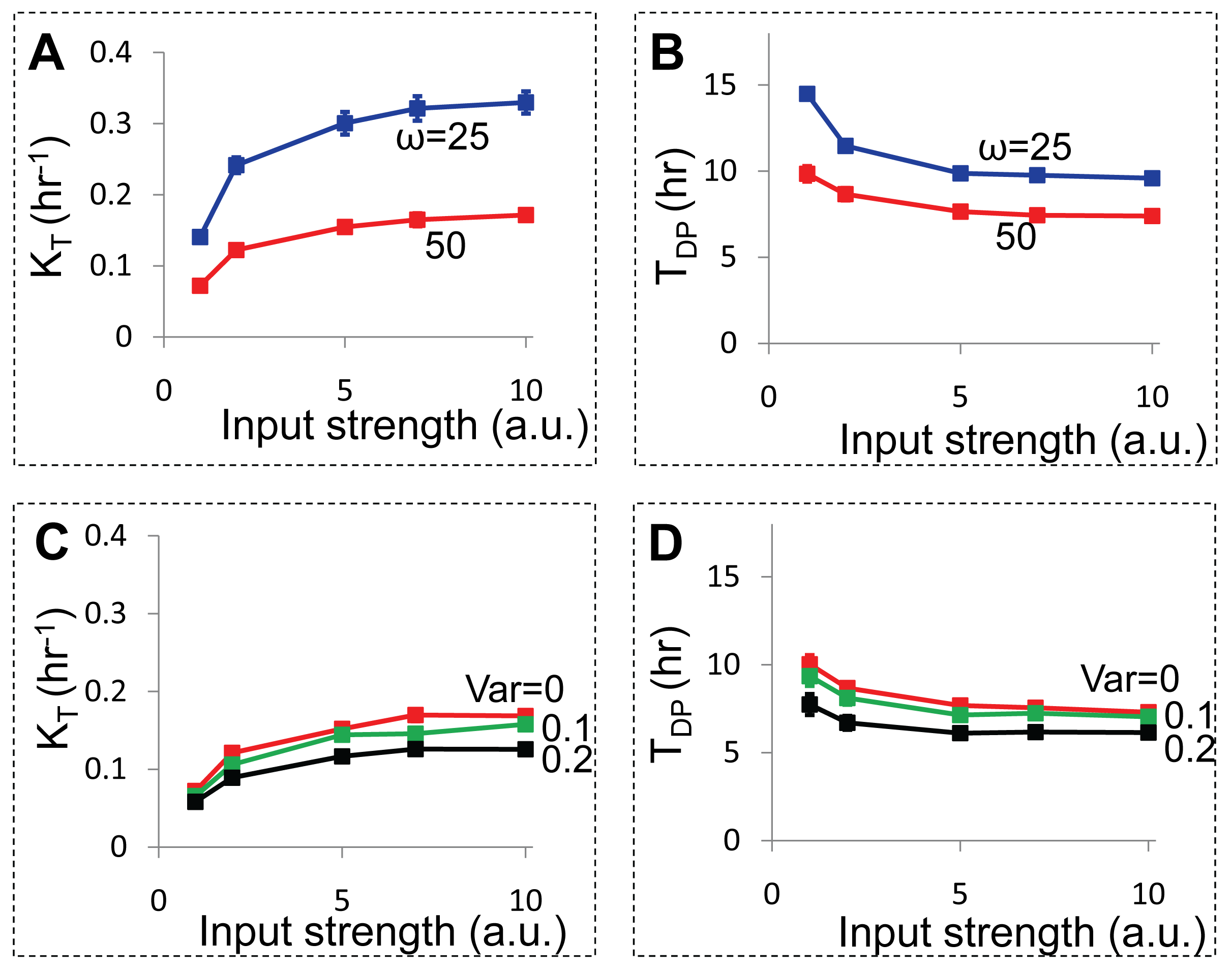

Supplement: Figure S8 — Variability in the initial conditions versus in the rates of the chemical reactions. The effects of variability in the initial conditions and in the rates of the chemical reactions were evaluated on the temporal dynamics of E2F activation. With all else the same, our simulation results predicted that transition rate (A) and time delay (B) would decrease significantly as ω was increased from 25 to 50. To describe variability in the initial condition, we assumed that the initial concentrations for Rb and the Rb-E2F complex were Gaussian-distributed with the mean being their base value and varying variance levels. At a fixed variance of extrinsic noise (ω = 50), our simulation results predicted that transition rate (C) and time delay (D) would decrease slightly with increasing variance of the initial conditions. Overall, the activation dynamics of E2F is much more sensitive to changes in extrinsic variability than those in the initial condition. (0.25 MB TIF) [file pbio.1000488.s008.tif]
