# Supplementary material for: Stochastic E2F Activation and Reconciliation of Phenomenological Cell-Cycle Models
Source: PLoS Biol. 2010 Sep 21;8(9):e1000488. doi: 10.1371/journal.pbio.1000488 (PMC2943438; doi:10.1371/journal.pbio.1000488)
Supplement: Text S1 — Model development. (0.27 MB DOC) [file pbio.1000488.s009.doc]

**Text S1. Model development**

**(Lee, Tae J. et al; Reconciling phenomenological cell-cycle models)**

**Model development**

The mathematical framework established in our previous work [1] consists of 7 ordinary differential equations (ODEs) and 23 parameters, as shown in Tables S1-S3. To develop a stochastic model based on the ODE framework, we adopted the chemical Langevin formulation [2,3] (see Equation 1 in the main text). We adjusted the units of the molecule concentrations and the parameters so that the molecules are expressed in molecular numbers. In addition, we made further adjustments to the free parameters and initial conditions (corresponding to quiescence), and introduced a new parameter (ω) based on the following constraints:

1. For the deterministic version of the model, the time required for E2F to reach half of its steady-state level is ~20 hours at 1% serum and 10 hours at 10% serum
2. The simulated distributions of E2F at the steady-state are consistent with the measured E2F distributions after 24 hours at both 0.5% and 1% serum concentrations: approximately 50% of the population is activated at 0.5% serum and approximately 90% is activated at 1% serum.
3. For a given serum concentration, once E2F is activated, noise cannot drive the system to become inactivated. This criterion serves as the high bound for the magnitude of the extrinsic noise
4. When E2F is activated, extrinsic noise is the predominant source of noise in the stochastic model. This criterion is based on previous observations that extrinsic noise typically contributes more to the overall noise in a cell population than intrinsic noise [4,5,6,7]. It serves as the low bound for the magnitude of the extrinsic noise

Using these constraints, we selected an optimal variance for ω, which determines the magnitude of extrinsic noise. To do this, we first calculated the intrinsic noise (η_int_ ) in E2F level at 10% serum, by carrying out stochastic simulations with ω = 0. We then chose a specific ω>0, and carried out stochastic simulations to obtain the total noise (η_tot_ ). The corresponding extrinsic noise can be calculated by using the relation η_tot_^2^ = η_int_^2^ + η_ext_^2^. By adjusting ω over a range of values, we determined an optimal value of 50. This value was used for all subsequent simulations, except for Figure S8 (where we examined the contribution of extrinsic variability to simulated stochastic dynamics). We note that the stochastic model will not violate the constraints outlined above for smaller serum concentrations, as long as E2F is in the activated state. For sufficiently small serum concentration, however, the intrinsic noise may become the dominant source of noise. In addition, we note that the overall noise in our model implementation is insensitive to our choice of base-line molecule numbers. For instance, for a larger molecule number, we would need to increase the variance of ω to ensure that the simulation results would satisfy the constraints outlined above. As such, our simulated stochastic dynamics of E2F and the phenomenological model parameters (e.g. T_DP_ or K_T_) would not be significantly altered by our selection of the base-line molecule numbers.

**Table S1. The deterministic model for the Myc/Rb/E2F pathway (adapted from Yao et al, 2008 [1]).**

| $\frac{d[M]}{dt}=\frac{k_{M}[S]}{K_{S}+[S]}-d_{M}[M]$ |
| --- |
| $\frac{d[E]}{dt}=k_{E}\left(\frac{[M]}{K_{M}+[M]}\right)\left(\frac{[E]}{K_{E}+[E]}\right)+\frac{k_{b}[M]}{K_{M}+[M]}+\frac{k_{P3}[CD][RE]}{K_{CD}+[RE]}+\frac{k_{P4}[CE][RE]}{K_{CE}+[RE]}-d_{E}[E]-k_{RE}[R][E]$ |
| $\frac{d[CD]}{dt}=\frac{k_{CD}[M]}{K_{M}+[M]}+\frac{k_{CDS}[S]}{K_{S}+[S]}-d_{CD}[CD]$ |
| $\frac{d[CE]}{dt}=\frac{k_{CE}[E]}{K_{E}+[E]}-d_{CE}[CE]$ |
| $\frac{d[R]}{dt}=k_{R}+\frac{k_{DP}[RP]}{K_{RP}+[RP]}-k_{RE}[R][E]-\frac{k_{P1}[CD][R]}{K_{CD}+[R]}-\frac{k_{P2}[CE][R]}{K_{CE}+[R]}-d_{R}[R]$ |
| $\frac{d[RP]}{dt}=\frac{k_{P1}[CD][R]}{K_{CD}+[R]}+\frac{k_{P2}[CE][R]}{K_{CE}+[R]}+\frac{k_{P3}[CD][RE]}{K_{CD}+[RE]}+\frac{k_{P4}[CE][RE]}{K_{CE}+[RE]}-\frac{k_{DP}[RP]}{K_{RP}+[RP]}-d_{RP}[RP]$ |
| $\frac{d[RE]}{dt}=k_{RE}[R][E]-\frac{k_{P3}[CD][RE]}{K_{CD}+[RE]}-\frac{k_{P4}[CE][RE]}{K_{CE}+[RE]}-d_{RE}[RE]$ |

Variables are:

S: growth signals (e.g. serum)

*M*: Myc

*E*: E2F

*CD*: CycD

*CE*: CycE

*R*: Rb

*RP*: Phosphorylated Rb

*RE*: RB-E2F complex.

Initial conditions:

[Rb]=0.4 µM, [RE]=0.25 µM, [M]=[E]=[CD]=[CE]=[RP]=0µM.

Underlying reactions for these ODEs are defined in Table S2.

Parameters are defined in Table S3.

**Table S2. Reaction kinetics for the model**

| **Reaction** | **Kinetics** | **Description and notes** |
| --- | --- | --- |
|  | $\frac{k_{M}[S]}{K_{S}+[S]}$ | Myc synthesis driven by growth signals (S) |
|  | $\frac{k_{CDS}[S]}{K_{S}+[S]}$ | CycD synthesis driven by growth signals |
|  | $\begin{matrix} k_{E}\left(\frac{[M]}{K_{M}+[M]}\right)\left(\frac{[E]}{K_{E}+[E]}\right) \\ +\frac{k_{b}[M]}{K_{M}+[M]} \end{matrix}$ | E2F synthesis by a synergy between Myc and E2F autocatalysis.  Since neither Myc nor E2F forms a homodimer, we assumed no cooperativity in gene activation mediated by these factors, and used the Hill coefficient of 1.0. Using Hill coefficient greater than 1.0 will not change the qualitative behavior of system dynamics |
|  | $\frac{k_{CE}[E]}{K_{E}+[E]}$ | CycE synthesis driven by E2F |
|  | $\frac{k_{CD}[M]}{K_{M}+[M]}$ | CycD synthesis driven by Myc |
| $*\xrightarrow{} [R]$ | $k_{R}$ | Constitutive Rb synthesis |
|  | $\frac{k_{P3}[CD][RE]}{K_{CD}+[RE]}+\frac{k_{P4}[CE][RE]}{K_{CE}+[RE]}$ | E2F dissociation from Rb-E2F complex by CycD- and CycE-mediated phosphorylation |
| $[E]+[R]\xrightarrow{} [RE]$ | $k_{RE}[R][E]$ | E2F titration by Rb via E2F-Rb complex formation |
|  | $\frac{k_{P1}[CD][R]}{K_{CD}+[R]}+\frac{k_{P2}[CE][R]}{K_{CE}+[R]}$ | Rb phosphorylation by CycD and CycE |
| $[RP]\xrightarrow{} [R]$ | $\frac{k_{DP}[RP]}{K_{RP}+[RP]}$ | Rb dephosphorylation |
| $[M]\xrightarrow{}*$ | $d_{M}[M]$ | Myc decay |
| $[E]\xrightarrow{}*$ | $d_{E}[E]$ | E2F decay |
| $[CE]\xrightarrow{}*$ | $d_{CE}[CE]$ | CycE decay |
| $[CD]\xrightarrow{}*$ | $d_{CD}[CD]$ | CycD decay |
| $[R]\xrightarrow{}*$ | $d_{R}[R]$ | Rb decay |
| $[RP]\xrightarrow{}*$ | $d_{RP}[RP]$ | Phosphorylated Rb decay |
| $[RE]\xrightarrow{}*$ | $d_{RE}[RE]$ | Rb-E2F complex decay |

**Table S3. Parameters for the model**

| **Rate constants** | | **Parameter values, sources, and notes** |
| --- | --- | --- |
| $k_{M}$ | 1 µM/hr | These values were adjusted together so that:  (1) The maximum E2F level is about 5-6 fold higher than the maximum CycD level (based on our experimental observations).  (2) The simulated E2F level will be around the corresponding Michaelis-Menten parameter (*K_E_*) in the deterministic simulations  (3) The deterministic time delay at 1% serum is approximately 20 hours and is halved at 10% serum  (4) Approximately 50% of the population is activated at 0.5% serum and approximately 90% is activated at 1% serum  (5) E2F activation is irreversible  (6) Extrinsic noise is the predominant source of noise in the stochastic model |
| ** $k_{E}$ | 0.15 µM/hr |
| ** $k_{CD}$ | 0.2 µM/hr |
| ** $k_{CDS}$ | 0.2 µM/hr |
| ** $k_{R}$ | 0.35 µM/hr |
| ** $k_{b}$ | 0.001 µM/hr |
| ** $k_{CE}$ | 0.5 µM/hr |
| ** $K_{S}$ | 0.3 µM |
| ** $k_{RE}$ | 30 / (µM * hr) |
| ω | 50 /hr |
| $d_{M}$ | 0.7/hr | Myc half-life = 60 min [8,9,10] |
| $d_{E}$ | 0.25/hr | E2F half-life = 2~3 hr [11] |
| $d_{CD}$ | 1.5/hr | CycD half-life = 25~30 min [12,13] |
| $d_{CE}$ | 1.5/hr | CycE half-life = 30 min [14,15] |
| $d_{R}$ | 0.06/hr | Rb half-life = 12 hours [16] |
| $d_{RP}$ | 0.06/hr | Assumed to be the same as $d_{R}$ |
| $d_{RE}$ | 0.03/hr | Rb-E2F half-life = 6 hours: The Rb-E2F complex assumed to be more stable than Rb alone [17] |
| $*k_{P1}$, $k_{P2}$ | 18/hr | Typical value phosphorylation rate constant [18] is 3600/hr |
| $k_{P3}$, $k_{P4}$ | 18/hr | Typical value phosphorylation rate constant [18] is 3600/hr |
| $k_{DP}$ | 3.6 µM/hr | Typical value for dephosphorylation rate assuming a constant phosphatase concentration [18] is 720 μM/hr |
| $K_{M}$ | 0.15 µM | Estimated based on measured Myc/Max –DNA dissociation constant [19] |
| $K_{E}$ | 0.15 µM | Assumed to be the same as *K_M_* |
| $K_{CD}$ | 0.92 µM | Experimentally measured [20,21] |
| $K_{CE}$ | 0.92 µM | Assumed to be the same as CycD |
| $K_{RP}$ | 0.01 µM | Typical value for Michaelis-Menten parameter for dephosphorylation [18] |

* Typical values of phosphorylation and dephosphorylation rate constants results in a stiff model, which drastically slows down the calculation. We have found that the overall dynamics is insensitive to the overall rates of the phosphorylation and dephosphorylation reactions, as long as they are balanced. Thus we have reduced the corresponding rate constants by 200 fold to speed up calculation. Proportionally increasing these parameters has no significant impact on the overall system dynamics.

** These ‘free’ parameters are modified from our previous deterministic model [1] to match simulations with experimental data.

**Citations:**

1. Yao G, Lee TJ, Mori S, Nevins JR, You LC (2008) A bistable Rb-E2F switch underlies the restriction point. Nature Cell Biology 10: 476-U255.

2. Gillespie DT (2000) The chemical Langevin equation. Journal of Chemical Physics 113: 297-306.

3. Lee TJ, Tan CM, Tu D, You LC (2007) Modeling cellular networks. Bioinformatics: An Engineering Case-Based Approach Chapter 6.

4. Raser JM, O'Shea EK (2004) Control of stochasticity in eukaryotic gene expression. Science 304: 1811-1814.

5. Raser JM, O'Shea EK (2005) Noise in gene expression: Origins, consequences, and control. Science 309: 2010-2013.

6. Rosenfeld N, Young JW, Alon U, Swain PS, Elowitz MB (2005) Gene regulation at the single-cell level. Science 307: 1962-1965.

7. Elowitz MB, Levine AJ, Siggia ED, Swain PS (2002) Stochastic gene expression in a single cell. Science 297: 1183-1186.

8. Sears R, Leone G, DeGregori J, Nevins JR (1999) Ras enhances Myc protein stability. Molecular Cell 3: 169-179.

9. Sears R, Nuckolls F, Haura E, Taya Y, Tamai K, et al. (2000) Multiple Ras-dependent phosphorylation pathways regulate Myc protein stability. Genes & Development 14: 2501-2514.

10. Yeh E, Cunningham M, Arnold H, Chasse D, Monteith T, et al. (2004) A signalling pathway controlling c-Myc degradation that impacts oncogenic transformation of human cells.[see comment]. Nature Cell Biology 6: 308-318.

11. Helin K (1998) Regulation of cell proliferation by the E2F transcription factors. Current Opinion in Genetics & Development 8: 28-35.

12. Diehl JA, Zindy F, Sherr CJ (1997) Inhibition of cyclin D1 phosphorylation on threonine-286 prevents its rapid degradation via the ubiquitin-proteasome pathway. Genes & Development 11: 957-972.

13. Sherr CJ, Roberts JM (1999) CDK inhibitors: positive and negative regulators of G1-phase progression. Genes & Development 13: 1501-1512.

14. Clurman BE, Sheaff RJ, Thress K, Groudine M, Roberts JM (1996) Turnover of cyclin E by the ubiquitin-proteasome pathway is regulated by cdk2 binding and cyclin phosphorylation. Genes & Development 10: 1979-1990.

15. Won KA, Reed SI (1996) Activation of cyclin E/CDK2 is coupled to site-specific autophosphorylation and ubiquitin-dependent degradation of cyclin E. EMBO Journal 15: 4182-4193.

16. Mihara K, Cao XR, Yen A, Chandler S, Driscoll B, et al. (1989) Cell cycle-dependent regulation of phosphorylation of the human retinoblastoma gene product. Science 246: 1300-1303.

17. Buchler NE, Gerland U, Hwa T (2005) Nonlinear protein degradation and the function of genetic circuits. PNAS 102: 9559-9564.

18. Kholodenko BN (2006) Cell-signalling dynamics in time and space. Nature Reviews Molecular Cell Biology 7: 165-176.

19. Park S, Chung S, Kim K-M, Jung K-C, Park C, et al. (2004) Determination of binding constant of transcription factor myc-max/max-max and E-box DNA: the effect of inhibitors on the binding. Biochimica et Biophysica Acta (BBA) - General Subjects 1670: 217-228.

20. Grafstrom RH, Pan W, Hoess RH (1999) Defining the substrate specificity of cdk4 kinase-cyclin D1 complex. Carcinogenesis 20: 193-198.

21. Pan W, Sun T, Hoess R, Grafstrom R (1998) Defining the minimal portion of the retinoblastoma protein that serves as an efficient substrate for cdk4 kinase/cyclin D1 complex. Carcinogenesis 19: 765-769.
