# Supplementary material for: Stochastic E2F Activation and Reconciliation of Phenomenological Cell-Cycle Models
Source: PLoS Biol. 2010 Sep 21;8(9):e1000488. doi: 10.1371/journal.pbio.1000488 (PMC2943438; doi:10.1371/journal.pbio.1000488)
Supplement: Text S2 — Calculation of metrics: TP and GC model parameters. (0.04 MB DOC) [file pbio.1000488.s010.doc]

**Text S2. Calculation of metrics: TP and GC model parameters**

**(Lee, Tae J. et al; Reconciling phenomenological cell-cycle models)**

**Calculation of metrics: TP model parameters (K_T_ and T_DP_) and GC model parameters ($\mathop{\text{r}}\limits^{-} ,$and σ):**

To estimate transition rate (K_T_) and time delay (T_DP_), we fitted the G0 exit curve with an exponential function (TP model):$\text{N(t)}=\text{N}_{0}\text{e}^{-\text{K}_{\text{T}}\text{*(t}-\text{T}_{\text{DP}}\text{)}}$; $\text{N(t}\leq\text{T}_{\text{DP}}\text{)}=\text{N}_{0}$, where N_0_ (=100%) is the initial percentage of cells in G0. We used least squares between the experimental data and estimation for curve-fitting. Similarly, we used least squares to fit the experimental data with the GC model: $\text{T}=1/\text{R}, \text{R}\sim N(\mathop{\text{r}}\limits^{-} \text{,} \text{σ}^{2})$, where R is a random variable normally distributed with the mean growth rate $\mathop{\text{r}}\limits^{-}$ and variance σ. T defines the distribution of times at which cells undergo the R-point traverse. The G0 exit curve was converted to the empirical cumulative distribution function (ECDF) for the R-point traverse. To facilitate parameter fitting, we assumed that the random variable R is approximately Gamma-distributed. Using least squares between the simulations/experimental data and the fitted GC model, we estimated GC model parameters from the ECDF. As a measure of precision, we used Monte-Carlo simulations to approximate standard deviation of these parameters. To do this, we generated a large number of datasets using the fitted model and their GC or TP parameters with their Monte-Carlo standard deviations were estimated.
